# Upper Midline Correction Using the Mesial-Distalslider

**DOI:** 10.3390/bioengineering11050450

**Published:** 2024-05-01

**Authors:** Maria Elena De Felice, Silvia Caruso, Maximilian Kueffer, Roberto Gatto, Benedict Wilmes

**Affiliations:** 1 Department of Life, Health and Environmental Sciences, University of L’Aquila, Piazzale Salvatore Tommasi 1, 67100 L’Aquila, Italy; silvia.cauruso@univaq.it (S.C.); roberto.gatto@univaq.it (R.G.); 2 Department of Orthodontics, University of Düsseldorf Heinrich Heine, Moorenstraße 5, 40225 Düsseldorf, Germany; maximilian.kueffer@med.uni-duesseldorf.de (M.K.); wilmes@med.uni-duesseldorf.de (B.W.)

**Keywords:** temporary anchorage devices, dento-alveolar asymmetries, model superimpositions, upper midline deviation, skeletal anchorage

## Abstract

Aim: The purpose of the present study is the three-dimensional (3D) analysis of molar and incisor movements that occur during the correction of the upper midline deviation by using the Mesial-Distalslider appliance. Materials and Methods: A total of 20 consecutive patients (12 women and 8 men; mean age 19.6 ± 11.1 years) were selected from the Orthodontic Department of Heinrich-Heine University of Düsseldorf. To correct the upper midline deviation (>2 mm), the patients were treated with asymmetric mechanics (mesialization on one side and distalization on the contralateral side) with the aid of Mesial-Distalslider. Dental casts were taken for each patient before (T0) and after the treatment (T1). The casts were 3D digitized and the models were superimposed on the palatal anterior region. Three-dimensional molar movements and sagittal incisor movements (proclination and retroclination) were assessed for T0 and T1. Results: At the end of the treatment, the total movements of the molars resulted in 4.5 ± 2.2 mm (antero-posterior direction), −0.4 ± 2.4 mm (transverse direction) and 0.3 ± 0.9 mm (vertical direction) on the mesialization side, and −2.4 ± 1.7 mm (antero-posterior direction), −0.5 ± 1.5 mm (transverse direction) and 0.2 ± 1.4 mm (vertical direction) on the distalization side. Incisor displacement was 0.9 mm ± 1.7 (mesialization side) and 0.6 mm ± 0.7 (distalization side). Conclusion: The Mesial-Distalslider appliance could be considered a valuable tool in orthodontic treatment for upper midline correction. Within the limits of a retrospective study, asymmetric molar movements appeared possible without clinically relevant anchorage loss.

## 1. Introduction

Midline deviation, one of the most challenging problems orthodontists encounter, may be observed in all types of malocclusions [1]. Very often, a lack of midline correspondence is observed between the upper and lower arches, or even if they correspond, they may not be symmetrically placed on the face [2]. “The upper dental midline is defined as the line passing through the contact points between the upper central incisors or the midpoint of the space between them in case of presence of the midline diastema and perpendicular to the upper occlusal plane” [3]. Arnett and Bergman [4] reported that the philtrum is the best reference point to be used in order to detect the facial midline.

Beyer et al. [5] noted that the majority of individuals can effectively discern midline discrepancies of 2 mm or more. This point of view is corroborated by Johnston et al. [6], who determined that a substantial number of orthodontists are inclined to detect dental and facial midline differences of 2 mm or more. An increase in the limit of acceptability was reported in the study of Ker et al. [7], who suggest that non-experts, of which one-third consider acceptable a threshold of 4.3 mm, perceive the highest acceptable threshold for upper dental midline deviation from the facial midline to be 2.9 mm.

In order to correct midline deviation, the first step in the diagnosis and treatment planning of all patients should be a correct differential diagnosis for the identification of these asymmetries, which are to be differentiated from a dental or a skeletal origin. It is only then that the clinician can make a valid decision concerning a correct treatment approach [8].

After establishing that the origin of the deviation is dental, it is necessary to identify whether the problem affects the upper and/or lower arch.

According to the study by Chrapla et al. [9], dental asymmetry, and subsequently, esthetic issues, can also arise from the differing dimensions of incisors and canines between the right and left sides. The ideal proportion between the left and right sides is observed in only 30% of individuals, with the maximum difference in lateral incisor width being 1.62 mm [10].

In our study, the examined patients presented with an upper midline deviation while the lower midline remained centered with the face. The aim of the present study was the three-dimensional (3D) analysis of molar and incisor movements that occur during the correction of the upper midline deviation using the Mesial-Distalslider appliance.

A predictable anchorage control is essential to preserve the lower midline and prevent undesirable buccal or lingual tipping of the maxillary and mandibular incisors during asymmetric mechanics [3,8]. In the last decade, there has been a surge in the use of mini-implants as temporary anchorage devices to provide reliable anchorage (TADs) [11,12,13,14]. These devices allow stable intraosseous anchorage [15].

The philosophy underlying skeletal anchorage is based on the idea that if the reactive forces can be absorbed by skeletal structures, tooth movement can be confined to the desired orthodontic movement, thus preventing undesirable reactive side effects.

The Mesial-Distalslider appliance, coupled with two mini-implants placed in the anterior area of the palate, has emerged as a highly recommended solution for this kind of treatment. This innovative appliance is designed to facilitate controlled mesial and distal tooth movement [16].

However, to the best of our knowledge, there are no quantitative assessments regarding the treatment effects associated with the Mesial-Distalslider appliance to correct the upper midline deviation.

## 2. Materials and Methods

The protocol of the present study was reviewed and approved by the ethics committee of Heinrich Heine University of Düsseldorf (No. 2023-2673). A total of 20 consecutive patients (12 women and 8 men with a mean age of 19.6 ± 11.1 years) were selected from the Department of Orthodontics at the University of Düsseldorf.

**Figure 1:**
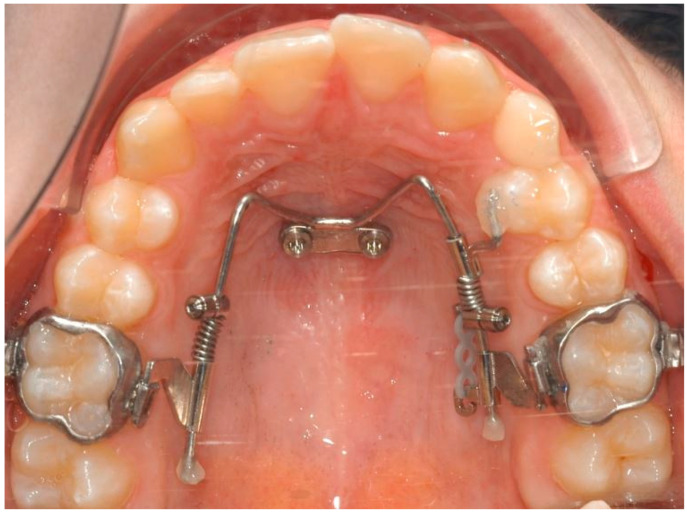
Clinical picture of the Mesial-Distalslider appliance.

Patients were recruited according to the following inclusion criteria: upper midline deviation > 2 mm with an asymmetric dental arch. The exclusion criteria were as follows: craniofacial syndromes, systemic diseases or comorbidities, moderate or severe periodontitis, and pharmacotherapeutic exposure with possible effects on bone metabolism. The diagnosis was carried out through an evaluation of all available diagnostic records such as pictures, orthodontic models, and radiographs. The patients were treated with the Mesial-Distalslider (TADMAN, Gunningen, Germany) during the first phase of their overall orthodontic treatment (Figure 1).

### 2.1. The Appliance

The Mesial-Distalslider is a mini-implant-borne appliance that uses the Beneslider mechanism for upper molar distalization [17,18] and the Mesialslider mechanism for upper molar mesialization [19], thus facilitating simultaneous distalization and mesialization movements [20,21]. The mesialization force is applied by using a nickel titanium closing spring (200 g), secured by an activation lock that is mesially pushed, while the distalization force is applied in children by using a nickel titanium open-coil spring of 240 g and of 500 g after eruption of the second molars with the activation lock distally pushed. Follow-up visits are planned every four to six weeks. If, after several months, excessive friction is noted along the mesialization side, elastic chains can be included. The premolars and canines will gradually move distally along the distalization side, leading to the development of small diastemas, while the premolars are pushed mesially on the opposite side. In some cases, the Mesial-Distalslider can be employed concurrently with bonded brackets or with aligners [22].

Dental casts were taken for each patient before (T0) and after the treatment (T1).

**Figure 2:**
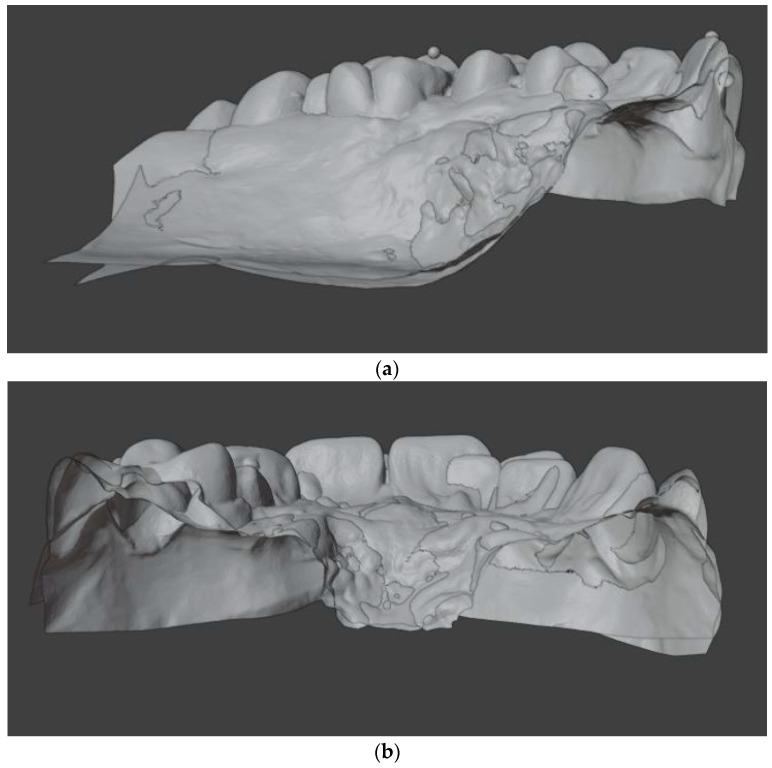
Cross-sectional view of the superimposed model: (**a**) sagittal plane; (**b**) transverse plane.

At first, the casts were 3D digitized using an intraoral scanner (3Shape Trios 3, Copenhagen, Denmark), followed by the superimposition of the models performed on the anterior region of the palate. The reconstruction and superimposition of the 3D digital models were carried out using the open-source 3D modeling software Blender (Version 3.6.2.) (Figure 2a,b).

All the processes were performed by two different investigators (M.E.D.F. and M.K.).

The T0 dental models were aligned to the occlusal plane defined by the mesiobuccal cusps of the first molars and the midpoint between the central incisors. Subsequently, a course alignment of the T1 models to the T0 models was performed manually. The fine alignment involved matching the palatal structures (rugae and palatal slope) using the iterative closest point (ICP) algorithm [23]. The alignment was restricted to an area in the anterior palate that is typically not affected by tooth movements [24,25,26,27,28,29].

**Figure 3:**
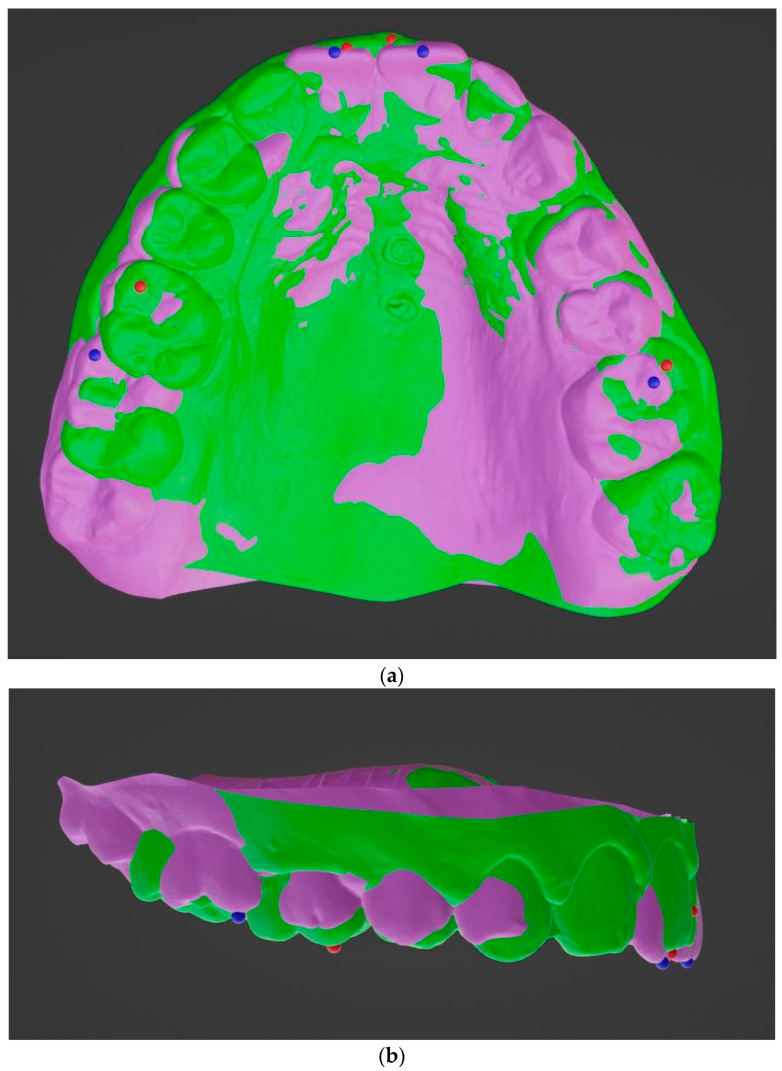
Superimposed pretreatment (purple) and post treatment (green) models: (**a**) occlusal view; (**b**) lateral view. Before treatment (blue dot) and at the end of the treatment (red dot).

Landmarks were identified at the mesiobuccal cusp of the upper first molars to analyze antero-posterior, transversal, and vertical movements along the y-, x-, and z-axes, respectively. In addition, the midpoint on the edge of the upper central incisors was designated to assess the proclination and retroclination along the y-axis. The reference points were marked with blue dots (T0) and with red dots (T1) on the digital models (Figure 3a,b).

The changes in the translation of each tooth between pretreatment (T0) and posttreatment (T1) models were measured in mm through lines connecting landmarks.

Movement in the positive direction along the y-axis indicated mesial movement, while positive values in the x- and z-axes indicated expansion and extrusive tooth movements, respectively. Negative values in the x-, y-, and z-axes indicated constriction, distal movement, and intrusion of the tooth, respectively. Prior to the orthodontic treatment, written informed consent was obtained from all of the participants.

### 2.2. Statistical Analysis

The statistical analysis was performed using the SPSS 20.0 statistical software package (SPSS Inc., Chicago, IL, USA). In order to assess the normality of the distribution of the data, the Shapiro–Wilk test was utilized. Continuous variables were presented as mean and standard deviation, and categorical variables were expressed as counts. The Spearman rho’s coefficient was employed to analyze the correlation between the variables of molar and incisor movements. All statistical analyses reported the probability that the values were two-tailed, and a *p* value < 0.05 was considered statistically significant.

### 2.3. Case Description

A 25-year-old male presented in our clinic for a consultation. He presented good general health and no systemic or congenital disease. His face, from a frontal view, appeared well-proportioned in the three-thirds with the upper midline deviated with respect to the face. From a lateral view, the patient showed a concave profile. Panoramic, lateral headfilm and dental cast records were taken. No clinical symptoms on articular examination were detected.

**Figure 4:**
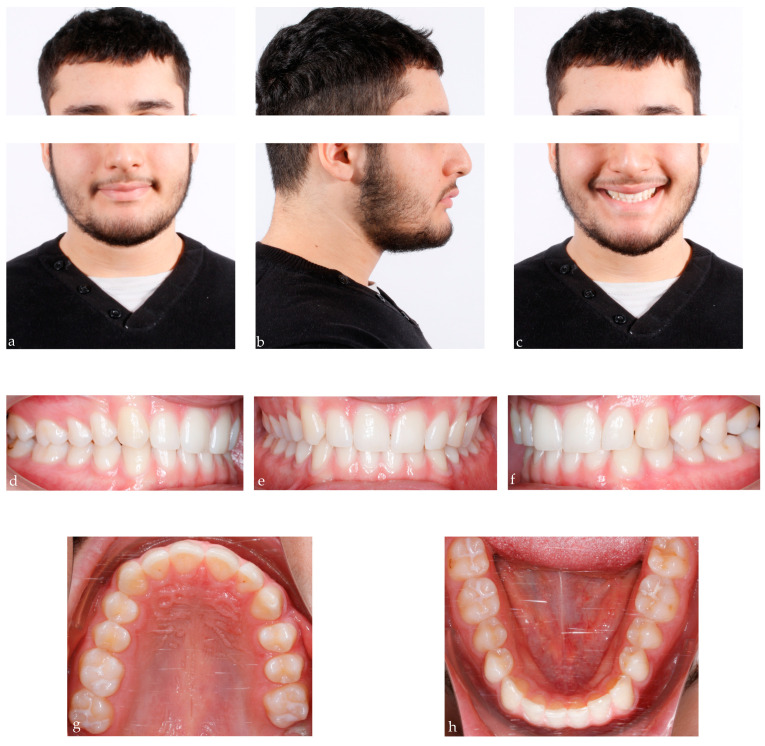

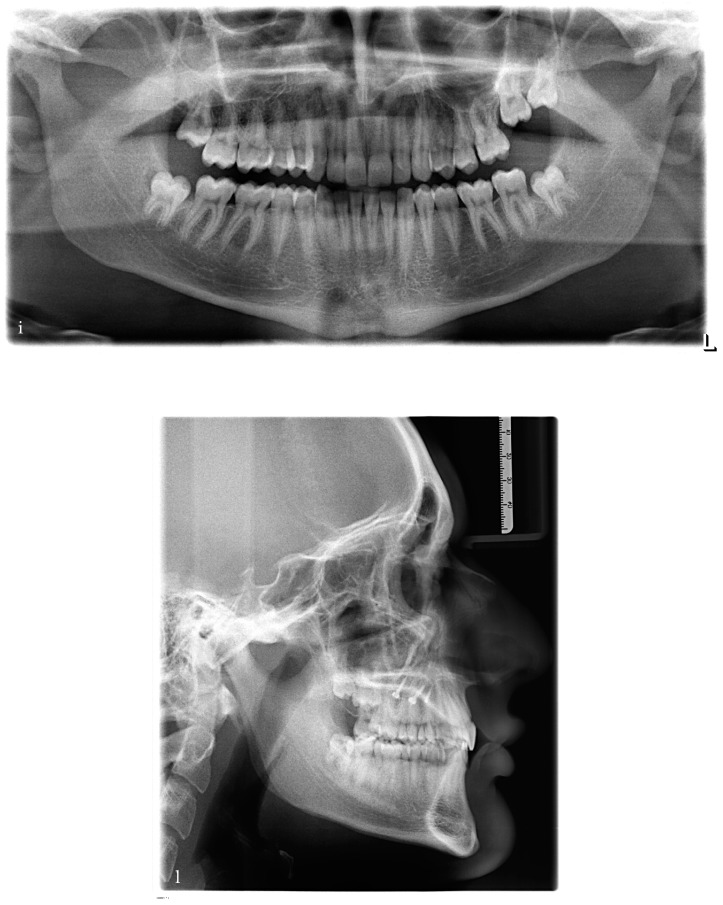
25-year-old male patient with skeletal class I, upper midline deviation, dental Class II subdivision; (**a**–**c**) extraoral pre-treatment pictures: lateral and frontal views; (**d**–**f**) intraoral pre-treatment pictures: lateral and frontal views; (**g**,**h**) occlusal views: upper and lower arch; (**i**,**l**): pre-treatment radiographs: panoramic and lateral cephalogram.

The cephalometric analysis showed a skeletal Class I with a tendency of class III (ANB 3.5°/Wits appraisal, −1.6 mm), hypodivergent pattern (SNˆGo-Gn 25.9°), and a retroclination of the upper and lower incisors. As regards intraoral examination, the patient revealed a Class II subdivision with a molar and canine Class II relationship on the right side and a molar and canine Class I relationship on the left side. He also presented normal overbite and overjet. (Figure 4)

### 2.4. Treatment Objectives and Alternatives

Two treatment options were considered, as follows:

- The first option, corresponding to our choice, involved the distalization of the upper right quadrant in order to reach the molar and canine Class I occlusion and to correct the upper midline deviation. At the same time, it involved the mesialization of the upper left quadrant in order to close the spaces opened during the treatment.
- The second option involved the extraction of the upper right first premolar in order to correct the Class II canine relationship and the deviation of the upper midline.

**Figure 5:**
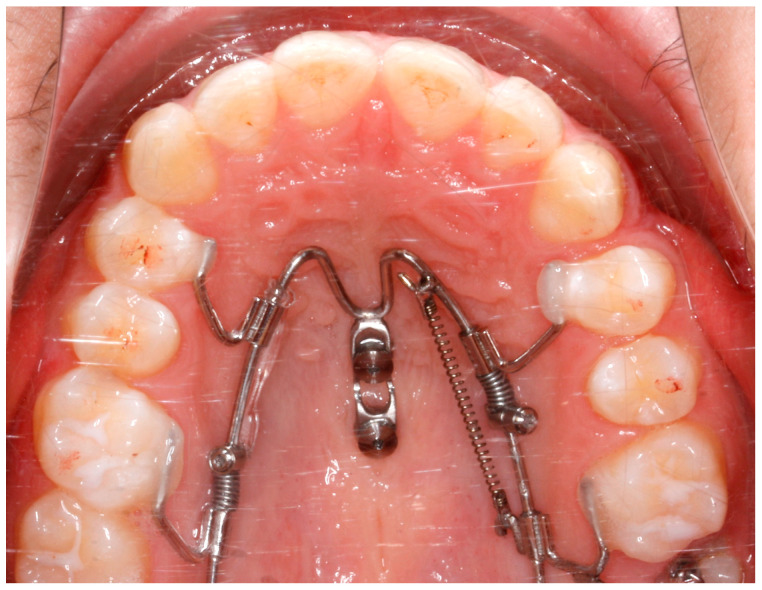
The Mesial-Distalslider appliance at the beginning of the treatment.

The first phase started with the use of the Mesial-Distalslider appliance (Figure 5). Two 2 × 9 mm mini-implants (Benefit system, PSM Medical Solutions, Gunningen, Germany) were placed in the anterior palate, and the Mesial-Distalslider appliance was placed and activated. The upper right first premolar was connected to the upper right first molar through an elastic chain for faster and more controlled movement during the distalization mechanics.

**Figure 6:**
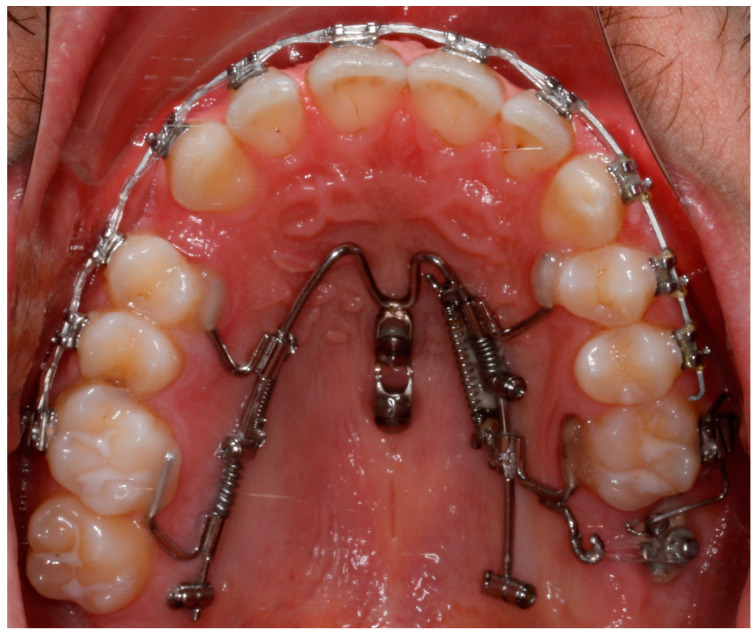
After 12 months of treatment with the Mesial-Distalslider.

After 12 months of treatment with the Mesial-Distalslider, full-arch fixed appliances were bonded in the upper arch after a Class I molar relationship was achieved and a space appeared mesial to the upper right first premolar (Figure 6).

Two archwires (0.016 NiTi and 0.016 × 0.022 NiTi) were utilized to progress to the working archwire, 0.018 × 0.025-inch stainless steel. At the end of the distalization, the device allowed for maximum molar anchorage in the first quadrant, moving the upper midline to the right side by using an elastic chain. Following the opening of the space mesial to the upper left canine, the Mesial-Distalslider was activated in the second quadrant to achieve mesialization of the entire quadrant without interfering with the correction of the upper midline. The patient easily adapted to the appliance and especially appreciated its esthetic appearance. After the complete correction, the brackets were removed. The total treatment time was about two years.

**Figure 7:**
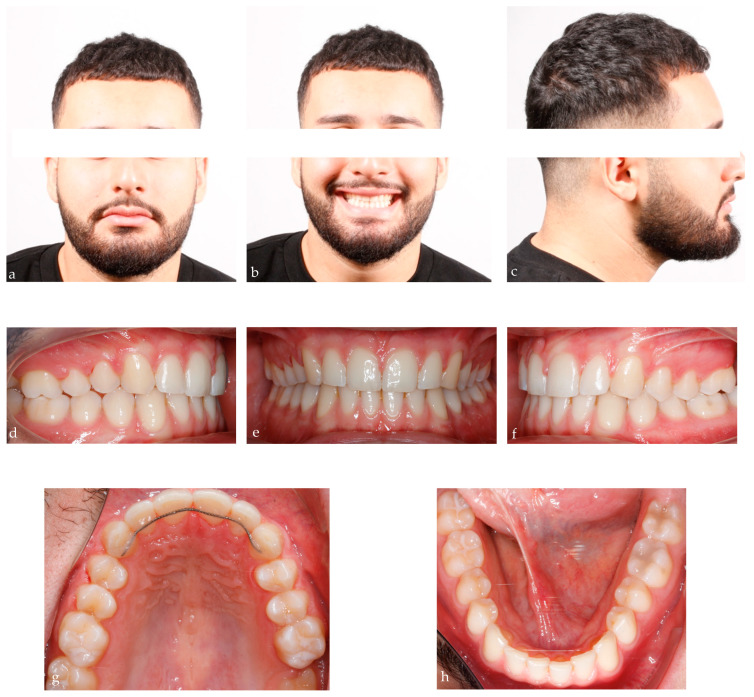

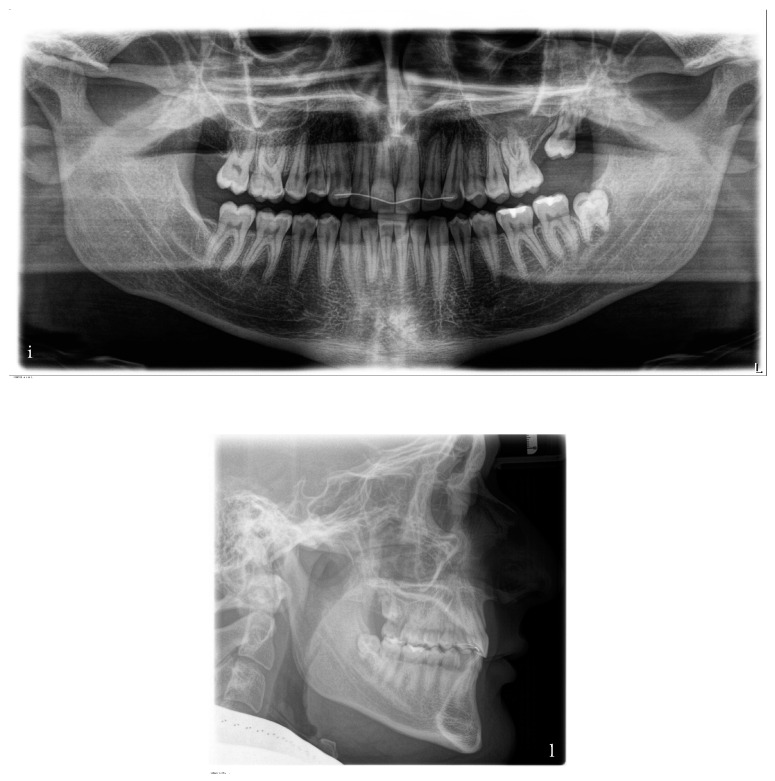
Final records of the patient after 28 months of treatment; (**a**–**c**) extraoral posttreatment pictures: lateral and frontal views; (**d**–**f**) intraoral posttreatment pictures: lateral and frontal views; (**g**,**h**) occlusal views: upper and lower arch; (**i**,**l**): posttreatment radiographs: panoramic and lateral cephalogram.

The post-treatment records of our patient showed a control in facial esthetics from the frontal perspectives, with a harmonious soft-tissue profile and an improvement in the smile. A molar and canine Class I occlusion was achieved, and the overbite and overjet were controlled. Coordination of the maxillary and mandibular midline was carried out, although the patient’s skeletal and esthetic characteristics remained (Figure 7).

## 3. Results

A total of 20 consecutive patients with ages ranging from 8 to 49 years were enrolled in this study. All patients requiring correction of the upper midline underwent treatment with the Mesial-Distalslider appliance. The average duration of the slider treatment was 12 months, and the entire orthodontic treatment was carried out in 28 months.

Total molar movements were 4.5 mm (SD 2.2) (antero-posterior), −0.4 mm (SD 2.4) (transversal), and 0.3 mm (SD 0.9) (vertical) on the mesialization side, and −2.4 mm (SD 1.7) (antero-posterior), −0.5 mm (SD 1.5) (transversal), and 0.2 mm (SD 1.4) (vertical) on the distalization side. Incisor displacement was 0.9 mm (SD 1.7) on the mesialization side and 0.6 mm (SD 0.7) on the distalization side (Table 1).

### 3.1. Mesialization Side

At T0, the mean values for molar positions were −2.7 (SD 4) (antero-posterior direction), 4 (SD 26) (transversal direction), and 5.8 (SD 3.1) (vertical direction). At T1, the mean values were 1.7 (SD 4.5) (antero-posterior direction), 3.6 (SD 24.1) (transversal direction), and 7.7 (SD 1.6) (vertical direction). Regarding incisor displacement, the mean value at T0 was 21.1 (SD 10.4) and at T1, 22.1 (SD 10.7) (antero-posterior direction). The mean differences (T1-T0) were 4.4 (SD 2.2) (antero-posterior direction), −0.4 (SD 2.4) (transversal direction), and 0.4 (SD 0.9) (vertical direction) for molar movements, and 0.9 (SD 1.7) for incisor displacement (Table 2).

### 3.2. Distalization Side

At T0, the mean values for molar positions were −0,3 (SD 6.3) (antero-posterior direction), −6.7 (SD 25.7) (transverse direction), and +7.1 (SD 1.5) (vertical direction). At T1, the mean values were −2.4 (SD 6.2) (antero-posterior direction), −7.3 (SD 27.1) (transversal direction), and 7.3 (SD 0.5) (vertical direction). Regarding incisor displacement, the mean value at T0 was 25.4 (SD 3.6) and at T1, it was 26 (SD 3.3) (antero-posterior direction). The mean differences (T1-T0) were −2.1 (SD 1.5) (antero-posterior direction), −0.5 (SD 1.5) (transversal direction), and 0.2 (SD 1.4) (vertical direction) for molar movements, and 0.6 (SD 0.7) for incisor displacement (Table 2).

Evaluating the differences among our variables, we observed that in both the mesialization and distalization sides, molars experienced a significant antero-posterior displacement (*p* = 0.000 and *p* = 0.006, respectively), with non-significant effects in the transverse and vertical directions.

Furthermore, the results indicate that minimal anchorage loss occurred at the incisal level during mesialization and distalization movements. The extent of distalization was minor, likely to be attributed to the subsequent mesialization of the molars after appliance removal. Hence, future consideration for enhanced anchorage control on the distalization side is warranted to preserve achieved results.

## 4. Discussion

The aim of this study was to analyze molar and incisor displacement during upper midline correction by using the Mesial-Distalslider appliance. A common indication for this type of appliance is an asymmetric upper arch with a midline deviation resulting from a unilateral missing tooth or a shift of the entire arch toward one side. The treatment of dental asymmetries is recognized as one of the most challenging processes in orthodontics. Moreover, the management of the space closure and the correction of the midline require optimal management of the anchorage. The term “orthodontic anchorage” was initially introduced by Edward Angle, and it is defined as a resistance to unwanted dental movements [30]. The universal desire among orthodontists is to have complete control over anchorage. In fact, the maintaining of proper anchorage control is crucial to achieving an ideal Class I relationship while conducting the asymmetrical movements. Systematic reviews and meta-analysis [31] demonstrated that the use of mini-implants is strongly recommended in case maximum anchorage is required. Moreover, their use is associated with a shorter treatment duration. Without implant anchorage, appliance complexity and biomechanics demands would be much greater. Moreover, these devices rely on primary stability (mechanical retention), which makes them immediately loadable, simple, and less invasive to remove [32,33].

As far as the treatment of midline deviation is concerned, no studies have been conducted to date to analyze three-dimensional molar movements and incisor displacement. In our study, changes in pre-treatment and post-treatment positions of landmarks were carefully examined by breaking up these variations into x-, y-, and z-coordinates, which represent tooth movements along the transverse, antero-posterior, and vertical directions. The measurements were carried out through the models’ superimpositions in the anterior region of the palate, recognized as a stable region in the literature [25,26,27,28,29]. Patients with dentoalveolar asymmetries often present some of the most biomechanically difficult situations. These cases may require various therapeutic options, including combined orthodontic–surgical treatments. In cases where patients decline orthodontic–surgical intervention, another approach can involve dental extractions, as reported in a clinical case by Rebellato et al. [34]. A similar approach was described by Ciavarella et al. [35], who successfully treated a patient with premolars extractions, coils, and laceback, avoiding the use of mini-implants. Treatment outcome was also possible due to patient compliance. Nevertheless, in extraction cases, two main situations have been identified: anchorage loss of molars during space closure after premolar extraction, and anchorage loss in the incisor or premolar region during distal movement of molars.

Conventional methods may lead to undesirable effects, such as molars constriction or expansion during mesialization and distalization mechanics. Additionally, along the mesialization side, there is a proclivity for incisors to retrocline, while a counter movement (proclination) occurs during distalization mechanics. The introduction of the skeletal anchorage has reduced or minimized this issue, given the counteracting force applied on the skeletal device [36].

In our study, the patients treated using the Mesial-Distalslider appliance exhibited successful mesial displacement of first molars by 4.5 mm (SD 2.2), with minimal transverse and vertical movements of −0.4 mm (SD 2.4) and 0.3 mm (SD 0.9), respectively. The mean upper incisor displacement was 0.95 mm (SD 1.7) on the mesialization side and 0.6 mm (SD 0.7) on the distalization side. These findings align with those presented by Becker et al. [23], who reported no displacements exceeding 0.5 mm in the transverse and vertical directions for both molars and incisors. Lai et al. [37] reported slight bodily intrusion during upper molar protraction with skeletal anchorage. On the contralateral side, a distal displacement of molars by −2.4 mm ± 1.7 was observed, with transverse and vertical movements of −0.5 mm ± 1.5 and 0.2 mm ± 1.4, which indicate constriction and extrusion, respectively. In our investigation, distal molar movement occurred with minimal transverse displacements. During distalization mechanics, several studies have reported mild molar intrusion ranging from 0.3 to 0.7 mm [38,39]. In every mechanics in which the line of action of the force is below the center of resistance, it takes an apical direction, and this intrusive effect could be shown [40]. The movement of the posterior dentition was achieved through ‘pushing’ mechanics of the permanent molars attached to the anterior and/or posterior part of the rail by using the NiTi coil springs which generate a force of 200/240 g. Three patients involved in this study did not exhibit significant movements along the three planes (sagittal, transverse, and vertical). Patients with the most significant antero-posterior movements were patient 13 and patient 17 who presented molar mesialization of 8.4 mm and 6.4 mm, respectively. In both cases, the expansion and extrusion of the moved molars were recorded. With regard to incisors, both patients exhibited incisal proclination. The greatest movement in the mesial and distal direction in the same arch was recorded in patient 2, in which both molars underwent an intrusion and an expansion. With this type of appliance, vertical movements of molars can be planned in the appliance design. The rail can be designed with an inclination that leads to molar intrusion or extrusion based on the desired outcome during distalization and mesialization movements. It allows for excellent three-dimensional management of the molars simultaneously without the need for multiple appliances. Furthermore, incisor displacement was 0.9 mm ± 1.7 on the mesialization side and 0.6 mm ± 0.7 on the distalization side. The results indicate that minimal anchorage loss occurred at the incisal level during mesialization and distalization movements. The incisal proclination during mesialization movement can often be attributed to two factors. The first factor is related to a combination of the palatal device with brackets, and it may be due to a friction between the arch and the brackets that leads to mesialization of the entire arch. In cases of incisal retroclination, simultaneous use of both can be helpful. The second factor may be due to a lack of space in the anterior sector or a lack of diastemas along the mesialization side. In cases where it is necessary to shift the upper midline, it would be advisable to start activation of the device from the distalization side so that the teeth on the mesialization side can smoothly traverse within the arch towards the desired direction. Moreover, the acceptance of adults due to the stability and reliability of mini-implant skeletal anchorage without compliance is being increased. An alternative option to correct midline is the use of intermaxillary elastics. In the literature, several methods recommend the use of intermaxillary elastics, but this approach carries the potential for further side effects in the opposite dental arch. In the study conducted by Lombardo et al. [41], the use of intermaxillary elastics had a double function: the anchorage was used to obtain simultaneous distalization of the molars and to correct the lower midline. Finally, two other case reports [42,43] described how to solve upper midline deviation when associated with maxillary arch deficiency by using different types of Hyrax expanders. Despite the success observed in these case reports, none of these studies considered a large sample of participants and no three-dimensional molar movements were analyzed through digital superpositions in any study. Considering our results, as the counteracting force is applied to the mini-implants in the anterior palate, side effects can be minimized through the utilization of the Mesial-Distalslider. Additionally, it becomes feasible to concurrently address multiple movements, ensuring effective three-dimensional control of the teeth targeted for displacement, and thereby diminishing potential side effects on adjacent structures that remain stationary. We propose the utilization of a partially osseointegrated mini-implant as it facilitates a more straightforward and precise application of force and vector. Since their retention is mostly mechanical, biomechanical factors must be considered indeed they have a higher chance of loosening due to torque or rotational forces under loading [44,45]. Many factors are decisive for their primary stability. These include the insertion angle, bone mineral density (BMD) of the receiver site, screw design, and number of screws used as an anchoring unit [46,47,48,49,50]. This type of implant also enables the application of rotational force without causing dislodgment, thus enhancing overall treatment accuracy. Nevertheless, from a statistical perspective, additional investigations with an expanded sample size should be taken into consideration.

## 5. Limitations

This study highlights two primary limitations. The first pertains to the absence of a control group, which is attributed to the lack of alternative devices capable of replicating the asymmetric mechanics of the Mesial-Distalslider. Future research might consider comparing mesialization movements using the unilateral Mesialslider and distalization movements using the unilateral Beneslider to address this limitation.

The second limitation is the lack of a sample size calculation. This issue arises because this is the inaugural study to explore this specific type of malocclusion, with no prior studies available for referencing when determining an appropriate sample size. Identifying an adequate sample size could significantly benefit future researchers in this field.

## 6. Conclusions

The use of Mesial-Distalslider for anchorage is an effective method for the upper midline correction. Furthermore, the biggest advantage of this technique is that patient cooperation is not required, and, in appropriate cases, it reduces the need for compensatory extractions on the distalization side and for dental implants on the mesialization side.

Within the limits of a retrospective study, the asymmetric molar movements appeared possible without clinically relevant anchorage loss.

## Figures and Tables

**Table 1:** Descriptive analysis; y (antero-posterior), x (transversal), z (vertical).

Mesialization Side								
Molar Movement (mm)	Axys	Mean	SD	Min	Max	25th	Median	75th
	y	4.5	2.2	0.0	8.4	3.1	4.9	5.8
	x	−0.4	2.4	−5.0	4.3	−1.4	−0.1	1.1
	z	0.3	0.9	−1.9	1.6	−0.2	0.4	1.2
Incisor Movement (mm)	y	0.9	1.7	−2.8	5.3	0.0	0.3	1.9
**Distalization side**								
Molar Movement (mm)	Axys	Mean	SD	Min	Max	25th	Median	75th
	y	−2.4	1.7	−4.6	−0.0	−4.2	−2.4	−0.5
	x	−0.5	1.5	−2.9	1.4	−1.9	−0.6	1.0
	z	0.2	1.4	−2.0	2.4	−0.5	−0.1	1.6
Incisor Movement (mm)	y	0.6	0.7	−0.4	1.8	0.1	0.3	1.3

**Table 2:** Mean paired difference among variables pre- and post-Mesial-Distalslider treatment.

	Mesialization Side				Distalization Side			
Molar Movements	T0	T1	Mean Differences	*p* Value	T0	T1	Mean Differences	*p* Value
**y (antero-posterior)**	−2.7 (SD 4)	1.7 (SD 4.5)	4.4 (SD 2.2)	0.000	−0.3 (SD 6.3)	−2.4 (SD 6.2)	−2.1 (SD 1.5)	0.006
**x (transversal)**	4 (SD 26)	3.6 (SD 24.1)	−0.4 (SD 2.4)	0.515	−6.7 (SD 25.7)	−7.3 (SD 27.1)	−0.5 (SD 1.5)	0.322
**z (vertical)**	5.8 (SD 3.1)	7.7 (SD 1,6)	0.4 (SD 0.9)	0.122	7.1 (SD 1.5)	7.3 (SD 0.5)	0.2 (SD 1.4)	0.698
**Incisors Displacement**	**T0**	**T1**	**Mean differences**	***p*** **value**	**T0**	**T1**	**Mean differences**	***p*** **value**
**y (antero-posterior)**	21.1 (SD 10.4)	22.1 (SD 10.7)	0.9 (SD 1.7)	0.042	25.4 (SD 3.6)	26 (SD 3.3)	0.6 (SD 0.7)	0.048

## Data Availability

Please contact the author for data requests.

## References

[1] Lewis P.D. (1976). The deviated midline. Am. J. Orthod..

[2] Shroff B., Lindauer S.J., Burstone C.J. (1997). Class II subdivision treatment with tip-back moments. Eur. J. Orthod..

[3] Daskalogiannakis J., World Federation of Orthodontists (2000). Glossary of Orthodontic Terms.

[4] Arnett G.W., Bergman R.T. (1993). Facial keys to orthodontic diagnosis and treatment planning. Part I Am. J. Orthod. Dentofac. Orthop..

[5] Beyer J.W., Lindauer J. (1998). Evaluation of dental midline position. Semin. Orthod..

[6] Johnston C.D., Burden D.J., Stevenson M.R. (1999). The influence of dental to facial midline discrepancies on dental attractiveness ratings. Eur. J. Orthod..

[7] Ker A.J., Chan R., Fields H.W., Beck M., Rosenstiel S. (2008). Esthetics and Smile Characteristics from the Layperson’s Perspective: A Computer-Based Survey Study. J. Am. Dent. Assoc..

[8] Burstone C.J. (1998). Diagnosis and treatment planning of patients with asymmetries. Semin. Orthod..

[9] Chrapla P., Paradowska-Stolarz A., Skoskiewicz-Malinowska K. (2022). Subjective and Objective Evaluation of the Symmetry of Maxillary Incisors among Residents of Southwest Poland. Symmetry.

[10] Gallão S., Ortolani C.L.F., Santos-Pinto A., Santos-Pinto L.A.M., Faltin K. (2010). Análise Fotográfica Da Simetria e Da Proporção Estética Dos Dentes Anteriores—Photographic Analysis of Symmetry and Aesthetic Proportion of Anterior Teeth. Rev. Inst. Ciênc..

[11] Costa A., Raffaini M., Melsen B. (1998). Miniscrews as orthodontic anchorage: A preliminary report. Int. J. Adult Orthodon Orthognath. Surg..

[12] Fritz U., Diedrich P., Kinzinger G., Al-Said M. (2003). The anchorage quality of mini-implants towards translatory and extrusive forces. J. Orofac. Orthop..

[13] Lim H.J., Choi Y.J., Evans C.A., Hwang H.S. (2011). Predictors of initial stability of orthodontic miniscrew implants. Eur. J. Orthod..

[14] Baumgaertel S. (2014). Temporary skeletal anchorage devices: The case for miniscrews. Am. J. Orthod. Dentofac. Orthop..

[15] Kuroda S., Sugawara Y., Deguchi T., Kyung H.-M., Takano-Yamamoto T. (2007). Clinical use of miniscrew implants as orthodontic anchorage: Success rates and postoperative discomfort. Am. J. Orthod. Dentofac. Orthop..

[16] Wilmes B., Nanda R., Nienkemper M., Ludwig B., Drescher D. (2013). Correction of upper-arch asymmetries using the Mesial-Distalslider. J. Clin. Orthod..

[17] Wilmes B., Nienkemper M., Ludwig B., Kau C.H., Pauls A., Drescher D. (2012). Esthetic Class II treatment with the Beneslider and aligners. J. Clin. Orthod..

[18] Wilmes B., Drescher D. (2010). Application and effectiveness of the Beneslider: A device to move molars distally. World J. Orthod..

[19] Wilmes B., Nienkemper M., Nanda R., Lübberink G., Drescher D. (2013). Palatally anchored maxillary molar mesialization using the Mesialslider. J. Clin. Orthod..

[20] Wilhelmy L., Willmann J.H., Tarraf N.E., Wilmes B., Drescher D. (2022). Maxillary space closure using a digital manufactured Mesialslider in a single appointment workflow. Korean J. Orthod..

[21] Wilmes B., Drescher D. (2023). CAD-CAM Workflows for Palatal TAD Anchored Appliances, Seminars in Orthodontics. Semin. Orthod..

[22] Wilmes B., Schwarze J., Vasudavan S., Drescher D. (2021). Maxillary Space Closure Using Aligners and Palatal Mini-Implants in Patients with Congenitally Missing Lateral Incisors. J. Clin. Orthod..

[23] Becker K., Wilmes B., Grandjean C., Drescher D. (2018). Impact of manual control point selection accuracy on automated surface matching of digital dental models. Clin. Oral. Investig..

[24] Almeida M.A., Phillips C., Kula K., Tulloch C. (1995). Stability of the palatal rugae as landmarks for analysis of dental casts. Angle Orthod..

[25] Hoggan B.R., Sadowsky C. (2001). The use of palatal rugae for the assessment of anteroposterior tooth movements. Am. J. Orthod. Dentofac. Orthop..

[26] Kim H.K., Moon S.C., Lee S.J., Park Y.S. (2012). Three-dimensional biometric study of palatine rugae in children with a mixed-model analysis: A 9-year longitudinal study. Am. J. Orthod. Dentofac. Orthop..

[27] Choi D.S., Jeong Y.M., Jang I., Jost-Brinkmann P.G., Cha B.K. (2010). Accuracy and reliability of palatal superimposition of three-dimensional digital models. Angle Orthod..

[28] Choi J.I., Cha B.K., Jost-Brinkmann P.G., Choi D.S., Jang I.S. (2012). Validity of palatal superimposition of 3-dimensional digital models in cases treated with rapid maxillary expansion and maxillary protraction headgear. Korean J. Orthod..

[29] Ashmore J.L., Kurland B.F., King G.J., Wheeler T.T., Ghafari J., Ramsay D.S. (2002). A 3-dimensional analysis of molar movement during headgear treatment. Am. J. Orthod. Dentofac. Orthop..

[30] Proffit W.R., Fields H.W., Larson B., Sarver D.M. (2018). Contemporary.

[31] Cornelis M.A., Scheffler N.R., De Clerck H.J., Tulloch J.F., Behets C.N. (2007). Systematic review of the experimental use of temporary skeletal anchorage devices in orthodontics. Am. J. Orthod. Dentofac. Orthop..

[32] Cha J.-Y., Kil J.-K., Yoon T.-M., Hwang C.-J. (2010). Miniscrew stability evaluated with computerized tomography scanning. Am. J. Orthod. Dentofac. Orthop..

[33] Ludwig B., Glasl B., Bowman S.J., Wilmes B., Kinzinger G.S., Lisson J.A. (2011). Anatomical guidelines for miniscrew insertion: Palatal sites. J. Clin. Orthod..

[34] Rebellato J. (1998). Asymmetric extractions used in the treatment of patients with asymmetries. Semin. Orthod..

[35] Ciavarella D., Maci M., Guida L., Cazzolla A.P., Muzio E.L., Tepedino M. (2023). Correction of Midline Deviation and Unilateral Crossbite Treated with Fixed Appliance. Case Rep. Dent..

[36] Chen Y.J., Chang H.H., Huang C.Y., Hung H.C., Lai E.H.H., Yao C.C.J. (2007). A retrospective analysis of the failure rate of three different orthodontic skeletal anchorage systems. Clin. Oral. Implant. Res..

[37] Lai E.H., Yao C.C., Chang J.Z., Chen I., Chen Y.J. (2008). Three-dimensional dental model analysis of treatment outcomes for protrusive maxillary dentition: Comparison of headgear, miniscrew, and miniplate skeletal anchorage. Am. J. Orthod. Dentofac. Orthop..

[38] Kilkis D., Celikoglu M., Nur M., Bayram M., Candirli C. (2016). Effects of zygoma-gear appliance for unilateral maxillary molar distalization: A prospective clinical study. Am. J. Orthod. Dentofac. Orthop..

[39] Oberti G., Villegas C., Ealo M., Palacio J.C., Baccetti T. (2009). Maxillary molar distalization with the dual-force distalizer supported by mini-implants: A clinical study. Am. J. Orthod. Dentofac. Orthop..

[40] Vilanova L., Castillo A.A.-D., Bellini-Pereira S.A., Henriques J.F.C., Janson G., Garib D., Patel M.P., Grec R.H.d.C., Yatabe M., Cevidanes L. (2023). Three-dimensional changes after maxillary molar distalization with a miniscrew-anchored cantilever. Angle Orthod..

[41] Lombardo L., Colonna A., Carlucci A., Oliverio T., Siciliani G. (2018). Class II subdivision correction with clear aligners using intermaxilary elastics. Prog. Orthod..

[42] Alcan T., Ceylanoğlu C. (2006). Upper midline correction in conjunction with rapid maxillary expansion. Am. J. Orthod. Dentofac. Orthop..

[43] Maspero C., Giannini L., Galbiati G., Farronato G. (2015). Modified transversal sagittal maxillary expander for correction of upper midline deviation associated with maxillary arch deficiency. Minerva Stomatol..

[44] Migliorati M., Signori A., Silvestrini-Biavati A. (2012). Temporary anchorage device stability: An evaluation of thread shape factor. Eur. J. Orthod..

[45] Pradal A., Nucci L., Derton N., De Felice M.E., Turco G., Grassia V., Contardo L. (2020). Mechanical Evaluation of the Stability of One or Two Miniscrews under Loading on Synthetic Bone. J. Funct. Biomater..

[46] Ferrillo M., Nucci L., Gallo V., Bruni A., Montrella R., Fortunato L., Giudice A., Perillo L. (2023). Temporary anchorage devices in orthodontics: A bibliometric analysis of the 50 most-cited articles from 2012 to 2022. Angle Orthod..

[47] Wilmes B., Ludwig B., Vasudavan S., Nienkemper M., Drescher D. (2016). The T-Zone: Median vs. Paramedian Insertion of Palatal Mini-Implants. J. Clin. Orthod..

[48] Han C.-M., Watanabe K., Tsatalis A.E., Lee D., Zheng F., Kyung H.-M., Deguchi T., Kim D.-G. (2019). Evaluations of miniscrew type-dependent mechanical stability. Clin. Biomech..

[49] Chang Z.-C.J., Chen Y.-J., Tung Y.-Y., Chiang Y.-Y., Lai E.H.-H., Chen W.-P., Lin C.-P. (2012). Effects of thread depth, taper shape, and taper length on the mechanical properties of mini-implants. Am. J. Orthod. Dentofac. Orthop..

[50] Lee J., Jeong Y., Pittman J., Deguchi T., Johnston W.M., Fields H.W., Kim D.-G. (2017). Primary stability and viscoelastic displacement of mini-implant system under loading. Clin. Biomech..

